# Editorial: Women in Inflammation Pharmacology: 2021

**DOI:** 10.3389/fphar.2022.973691

**Published:** 2022-07-11

**Authors:** Paola Patrignani

**Affiliations:** Department of Neuroscience, Imaging and Clinical Sciences, and Center for Advanced Studies and Technology (CAST), “G. d'Annunzio” University, Chieti, Italy

**Keywords:** inflammation pharmacology, 2021, women in inflammation pharmacology, gender equality, COVID—19, pain

According to UNESCO, less than 30% of researchers worldwide are women. Long-standing biases and gender stereotypes are discouraging girls and women from science-related fields, particularly STEM (science, technology, engineering, and mathematics research). However, science and gender equality are essential to ensure sustainable development, as highlighted by UNESCO. To change traditional mindsets, gender equality must be promoted, stereotypes defeated, and girls and women should be encouraged to pursue STEM careers. Therefore, Frontiers in Pharmacology has promoted the publication of the work of women scientists across all areas of Inflammation Pharmacology. Four excellent manuscripts have been published on the research topic “Women in Inflammation Pharmacology: 2021″ by

1) Santoso et al. who explored the transcriptional regulators of inflammatory cytokines involved in the COVID-19 cytokine release syndrome (CRS) to identify candidate transcription factors (TFs) for therapeutic targeting using approved drugs;
2) Hellmuth et al. who studied the effects of a series of (nitro-fatty acids) NFA derivatives regarding their modulatory potency and efficacy on the NF-κB (nuclear factor kappa B) signaling inhibition, induction of Nrf-2 (nuclear factor erythroid 2-related factor 2) gene expression, sEH (soluble epoxide hydrolase), LO (lipoxygenase), and COX-2 (cyclooxygenase-2) inhibition, and their cytotoxic effects on colorectal cancer cells;
3) Mui et al. who reviewed the biological functions of extracellular annexins and their possible therapeutic applications in the treatment of various disease conditions, especially sepsis, and novel diseases such as SARS-CoV-2 infection;
4) Valdrighi et al. who summarized and discussed the emerging evidence of sex differences regarding innate immunity in osteoarthritis (OA) pain to provide evidence on the development of alternative pain relief therapies targeting innate immunity that consider sex differences.

Uncontrolled acute inflammation and its progression to chronic inflammation are associated with the development of many human diseases (Dovizio et al., 2017; Furman et al., 2019; Ballerini et al., 2022). Despite anti-inflammatory agents such as nonsteroidal anti-inflammatory drugs (NSAIDs) (Patrignani and Patrono, 2015), glucocorticoids, antileukotrienes (Scadding and Scadding, 2010), and biologic drugs (Cavalli et al., 2020) are available and have shown efficacy in several clinical inflammation-associated diseases; they are linked with side effects and intrasubject variability in their responses (Bruno et al., 2014). Notably, the possible influence of sex in their responses has not been clarified yet (Farkouh et al., 2021).

Coronavirus disease 2019 (Covid-19) is still a global health problem (World Health Organization, 2021). Only glucocorticoids are known to improve survival among severely ill patients, supporting the concept that an excessive host inflammatory response is responsible for much of the serious illness and death from Covid-19 (The WHO Rapid Evidence Appraisal for COVID-19 Therapies Working Group, 2020).

A paper published by Santoso et al. on this Research Topic verified the capacity of approved drugs to target transcriptional regulators of cytokines associated with the CRS. The authors identified the potential therapeutic effect of 10 drugs on the expression of cytokines upregulated in COVID-19 patients. Moreover, 25 drug combinations were tested, and some showed promising synergistic efficacy in downregulating the expression of inflammatory cytokines. Whether these findings are effective in SARS-CoV-2 infection requires additional studies.

An approach to fight viral sepsis as in COVID-19 has been proposed by Mui et al. using annexins. The annexin superfamily contains over 1,000 proteins found in over 65 different species of plants and animals (Gerke and Moss, 2002). The authors report the evidence suggesting the potential of Annexins use for patients suffering from sepsis and COVID-19-induced sepsis or coagulopathy and ischemic-reperfusion injury. However, confirmation of these benefits is required in human trials.

NFAs are of interest as scaffolds for designing future anti-inflammatory drugs. In fact, NFAs have shown anti-inflammatory, cytoprotective, and anti-tumorigenic effects in different animal models of disease and safety in phase I clinical trials (Piesche et al., 2020). Here, Hellmuth et al. addressed the relationship between the chemical structure of NFA and their biological activity on distinct signaling pathways or selected target proteins and identified the lead compound 9NOA.

Finally, Valdrighi et al. reviewed the emerging evidence of sex differences regarding innate immunity in OA pain. The clarification of this issue will allow the development of more effective pain treatments in both women and men, thus avoiding the exposure of nonresponder individuals to the possible toxicity of drugs. In the future, more research on the analgesic effects of drugs in OA trials should be conducted on women since most data of efficacy were preferentially conducted on male subjects.
